# Draft Genome Sequences of Bacillus licheniformis and Bacillus paralicheniformis Strains Isolated from Irish Skim Milk Powder

**DOI:** 10.1128/mra.00137-23

**Published:** 2023-06-06

**Authors:** Antonio Lourenco, Fang Li, Narciso M. Quijada, Geraldine Duffy, John T. Tobin, Francis Butler, Kieran Jordan, Triona O’Brien

**Affiliations:** a Food Bioscience Department, Teagasc Food Research Centre, Moorepark, Fermoy, County Cork, Ireland; b Food Safety Department, Teagasc Food Research Centre, Moorepark, Fermoy, County Cork, Ireland; c Food Chemistry and Technology Department, Teagasc Food Research Centre, Moorepark, Fermoy, County Cork, Ireland; d School of Biosystems and Food Engineering, Agriculture and Food Science Centre, University College Dublin, Belfield, Dublin, Ireland; e Department of Microbiology and Genetics, Institute for Agribiotechnology Research (CIALE), University of Salamanca (USAL), Salamanca, Spain; University of Rochester School of Medicine and Dentistry

## Abstract

Nineteen Bacillus licheniformis strains and four strains of the closely related species Bacillus paralicheniformis were isolated from a variety of Irish medium-heat skim milk powders. The draft genome sequences of these 23 isolates provide valuable genetic data for research work relevant to dairy products and process development. The isolates are available at Teagasc.

## ANNOUNCEMENT

Bacillus licheniformis and Bacillus paralicheniformis are thermoresistant Gram-positive spore-forming bacteria that are commonly isolated from various dairy products, including milk powders, as well as farm environments (1–4). Neither species is clearly defined as pathogenic, but both have been associated with pathogenicity (5), food spoilage, and food poisoning incidents due to their toxin-producing ability.

B. licheniformis and *B. paralicheniformis* grow in a wide range of temperatures and facultative anaerobic conditions, making them difficult to control at all dairy processing steps (6).

In a previous screening study of the microbiota of Irish skim milk powders targeting spore-forming bacteria (4), 23 strains were isolated from different industrial sources. Those strains were obtained using one of two heat resistance treatments (80°C/10 min or 100°C/30 min), followed by incubation under aerobic or anaerobic conditions. Of the 23 strains, 11 were high heat-resistant spores that would be potentially challenging to inactivate during thermal processing technologies, such as high-temperature short-time (HTST) pasteurization, ultrahigh temperature (UHT) treatment, or spray drying (7–9). Enumeration, isolation, and purification of strains were performed as previously described (4).

The bacterial isolates are available at the Teagasc Food Safety Department (Moorepark) bacterial culture collection, where they are stored at −80°C.

For whole-genome sequencing, the isolates were incubated for 18 h at isolation temperature from a cryobead in 10 mL of brain heart infusion (BHI) broth. DNA was extracted from 2 mL of bacterial culture using the DNeasy UltraClean microbial kit (Qiagen, Venlo, Netherlands), following the manufacturer’s instructions, and quantified using a Qubit 2.0 fluorometer (Invitrogen, CA, USA), with the Qubit double-stranded DNA (dsDNA) high-sensitivity (HS) assay (Thermo Fisher Scientific), according to the supplier’s instructions. Library preparation and quantification were performed using the Nextera XT DNA sample preparation kit (Illumina, CA, USA) and the Qubit dsDNA HS assay, respectively. Library size distribution and quality were assessed using the 2200 TapeStation system (Agilent Technologies, CA, USA). Manual library normalization was performed, and the libraries were pooled with 5 μL of each 2.0 nM normalized library. Subsequently, 600 μL of a 12 pM library was made with a 1% PhiX control spike-in. Sequencing was performed on an Illumina MiSeq platform using the MiSeq reagent kit v3, yielding 300-bp paired-end reads.

Whole-genome sequencing analyses were performed using the TORMES v1.3.0 pipeline (10) with default software and parameters over the raw sequencing data, unless otherwise specified. The quality filtering process was performed using Trimmomatic v0.40 (11) (minimum read quality, 25; minimum read length, 50 bp). *De novo* genome assembly was carried out using SPAdes v3.15.2 (12) (minimum length of each contig to be kept in the genome after assembly, 200 bp). The quality and statistics of the assemblies were assessed using QUAST (13). Taxonomic identification of the sequencing data was performed using Kraken v2 (14). Genome annotation was conducted using Prokka v1.14.6 (15), and antimicrobial resistance screening was performed using BLASTN (16) built into ABRicate (Seemann; https://github.com/tseemann/abricate) and the ResFinder database (17).

The assembly statistics and main genome features are shown in Table 1.

**TABLE 1:** General features of the genomes sequenced^*a*^

Strain	Family (% of reads)	Genus (% of reads)	Species (% of reads)	No. of reads	No. of contigs (>200 bp)	Genome length (bp)	Largest contig (bp)	Contig *N*_50_ (bp)	GC content (%)	Sequencing depth (×)	No. of CDSs	No. of rRNAs	No. of tRNAs	No. of tmRNAs	Antimicrobial resistance screening^*a*^				GenBank accession no.	SRA accession no.
															Gene	Coverage (%)	% identity (GenBank accession no. of closest match)	Product		
DPTC10_3168^*b*^	*Bacillaceae* (98.89)	*Bacillus* (98.81)	Bacillus licheniformis (18.88)	2,286,138	62	4,215,112	794,208	424,763	46.13	156	4,283	12	79	1					JARAFA000000000	SRR24425836
DPTC11_3245^*c*^	*Bacillaceae* (99.55)	*Bacillus* (99.49)	Bacillus licheniformis (14.87)	1,380,580	70	4,191,660	1,288,030	303,315	46.16	96	4,262	10	81	1	*erm*(D)_3	100	100 (M77505)	*erm*(D)	JARAFB000000000	SRR24425835
DPTC1_3328^*c*^	*Bacillaceae* (99.51)	*Bacillus* (99.45)	Bacillus licheniformis (14.06)	3,087,310	683	4,625,408	566,540	420,093	45.99	191	4,575	12	79	1					JARAFC000000000	SRR24425824
DPTC13_3376^*c*^	*Bacillaceae* (99.52)	*Bacillus* (99.46)	Bacillus licheniformis (15.72)	1,224,300	54	4,263,609	744,518	444,109	45.84	83	4,353	12	79	1					JARAFD000000000	SRR24425820
DPTC14_3226^*b*^	*Bacillaceae* (99.24)	*Bacillus* (99.18)	Bacillus paralicheniformis (90.52)	2,166,970	58	4,437,393	782,344	478,415	45.6	141	4,408	12	81	1	*erm*(D)_1	100	99.9 (M29832)	*erm*(D)	JARAFE000000000	SRR24425819
DPTC15_3285^*b*^	*Bacillaceae* (98.27)	*Bacillus* (98.19)	Bacillus paralicheniformis (88.19)	2,409,736	91	4,527,348	844,392	242,197	45.8	151	4,542	17	82	1	*erm*(D)_1	100	99.8 (M29832)	*erm*(D)	JARAFF000000000	SRR24425818
DPTC16_3330^*c*^	*Bacillaceae* (99.05)	*Bacillus* (98.96)	Bacillus paralicheniformis (90.51)	5,371,688	56	4,454,351	900,159	620,291	45.65	346	4,427	12	80	1	*erm*(D)_1	100	98.5 (M29832)	*erm*(D)	JARAFG000000000	SRR24425817
DPTC17_3130^*b*^	*Bacillaceae* (99.55)	*Bacillus* (99.49)	Bacillus licheniformis (18.73)	2,272,542	93	4,360,113	605,381	368,842	45.83	152	4,467	11	79	1					JARAFH000000000	SRR24425816
DPTC18_3151^*b*^	*Bacillaceae* (99.56)	*Bacillus* (99.48)	Bacillus licheniformis (13.23)	2,199,744	43	4,124,108	664,416	378,984	46.16	155	4,170	11	79	1					JARAFI000000000	SRR24425815
DPTC19_3196^*c*^	*Bacillaceae* (99.27)	*Bacillus* (99.17)	Bacillus licheniformis (20.59)	3,415,084	46	4,264,904	1,295,074	989,612	45.83	232	4,360	12	79	1	*erm*(D)_3	99.2	95.7 (M77505)	*erm*(D)	JARAFJ000000000	SRR24425814
DPTC20_3156^*c*^	*Bacillaceae* (99.00)	*Bacillus* (98.85)	Bacillus licheniformis (10.35)	1,405,064	126	4,197,465	172,307	81,810	46.13	85	4,241	11	79	1					JARAFK000000000	SRR24425834
DPTC21_3181^*b*^	*Bacillaceae* (99.59)	*Bacillus* (99.53)	Bacillus licheniformis (15.20)	2,582,824	65	4,154,884	1,288,031	303,300	46.22	182	4,203	10	79	1	*erm*(D)_3	100	100 (M77505)	*erm*(D)	JARAFL000000000	SRR24425833
DPTC22_3213^*b*^	*Bacillaceae* (99.45)	*Bacillus* (99.38)	Bacillus licheniformis (13.30)	2,636,076	68	4,186,141	1,278,173	511,798	46.16	181	4,254	12	79	1	*erm*(D)_3	100	100 (M77505)	*erm*(D)	JARAFM000000000	SRR24425832
DPTC23_3165^*c*^	*Bacillaceae* (99.34)	*Bacillus* (99.26)	Bacillus licheniformis (18.45)	6,270,052	163	4,417,735	757,750	383,392	45.77	407	4,488	12	80	1	*erm*(D)_3	100	100 (M77505)	*erm*(D)	JARAFN000000000	SRR24425831
DPTC2_3370^*b*^	*Bacillaceae* (98.20)	*Bacillus* (98.12)	Bacillus paralicheniformis (89.26)	2,540,672	1046	4,900,371	263,241	35,802	45.7	149	4,701	11	88	1	*erm*(D)_3	93.9	98.5 (M77505)	*erm*(D)	JARAFO000000000	SRR24425830
DPTC24_3211^*c*^	*Bacillaceae* (99.32)	*Bacillus* (99.22)	Bacillus licheniformis (20.04)	1,586,480	67	4,223,220	720,397	214,880	46.15	108	4,585	12	80	1					JARAFP000000000	SRR24425829
DPTC3_3122^*c*^	*Bacillaceae* (98.93)	*Bacillus* (98.85)	Bacillus licheniformis (19.36)	3,297,568	126	4,271,439	1,076,265	438,762	46.29	224	4,348	11	79	1					JARAFQ000000000	SRR24425828
DPTC4_3301^*b*^	*Bacillaceae* (99.51)	*Bacillus* (99.44)	Bacillus licheniformis (25.93)	2,711,002	48	4,623,884	1,317,640	405,549	45.31	170	4,819	11	81	1					JARAFR000000000	SRR24425827
DPTC5_3247^*b*^	*Bacillaceae* (95.88)	*Bacillus* (95.81)	Bacillus licheniformis (14.00)	2,337,352	53	4,470,899	798,383	424,749	45.39	146	4,658	11	86	1					JARAFS000000000	SRR24425826
DPTC6_3313^*b*^	*Bacillaceae* (99.54)	*Bacillus* (99.47)	Bacillus licheniformis (19.87)	1,974,030	98	4,451,921	604,659	252,913	45.73	127	4,606	12	80	1					JARAFT000000000	SRR24425825
DPTC7_3265^*c*^	*Bacillaceae* (99.21)	*Bacillus* (99.11)	Bacillus licheniformis (21.04)	4,039,056	89	4,219,597	1,267,930	1,084,832	46.05	278	4,252	13	79	1	*erm*(D)_3	99.2	95.7 (M77505)	*erm*(D)	JARAFU000000000	SRR24425823
DPTC8_3316^*c*^	*Bacillaceae* (98.98)	*Bacillus* (98.90)	Bacillus licheniformis (19.77)	2,215,296	50	4,203,997	1,430,180	477,115	46.06	152	4,266	11	79	1	*erm*(D)_3	99.2	95.7 (M77505)	*erm*(D)	JARAFV000000000	SRR24425822
DPTC9_3308^*b*^	*Bacillaceae* (98.80)	*Bacillus* (98.72)	Bacillus licheniformis (19.63)	1,682,964	48	4,295,936	1,838,793	1,097,076	45.91	102	4,415	12	81	1					JARAFW000000000	SRR24425821

a The *erm*(D) gene confers resistance to erythromycin, lincomycin, clindamycin, quinupristin, pristinamycin IA, and virginiamycin S. Antimicrobial resistance screening of the genomes was conducted against ResFinder (17).

b Spore heat resistance treatment of 80°C for 10 min.

c Spore heat resistance treatment of 100°C for 30 min (highly heat-resistant spores).

### Data availability.

The genome sequences of all 23 strains are publicly available at NCBI GenBank under BioProject accession number PRJNA913261 (“Spore-forming bacteria in skim milk powders”). The GenBank and Sequence Read Archive (SRA) accession numbers are listed in Table 1. This announcement represents the first version of all 23 genomes.
